# Adherence to single inhaler triple therapy and digital inhalers in Chronic Obstructive Pulmonary Disease: a literature review and protocol for a randomized controlled trial (TRICOLON study)

**DOI:** 10.1186/s12890-024-03044-3

**Published:** 2024-07-04

**Authors:** Liz J. A. Cuperus, Job van der Palen, Arnoud Aldenkamp, Astrid van Huisstede, Erik W. M. A. Bischoff, Job F. M. van Boven, Folkert Brijker, Stephan Dik, Jeroen A. J. M. van Excel, Martijn Goosens, Peter Th. W. van Hal, Jolanda C. Kuijvenhoven, Lisette I. Z. Kunz, Erwin C. Vasbinder, Huib A. M. Kerstjens, Johannes C. C. M. in ’t Veen, the TRICOLON study group, Marjo van der Poel, Marijke Amelink, Anke Rol, Jennifer de Graaf, Petra Hirmann, Fleur van Tour, Elly Jordens, Lydia Alfing, Gerda Lenderink, Thecla Rupert, Truus Rietveld, Jasmijn van Campen, Jantine de Bruijn, Janice ter Burg, Walter van Litsenburg, Len Knoops, Margot Eggermont-Schilt, Manon de Waard-Heijligers, Ilonka Paalvast-Schouten, Sarah van Oord

**Affiliations:** 1 Pulmonology Department, Franciscus Gasthuis and Vlietland, Rotterdam, the Netherlands; 2 Pulmonology Department, University of Groningen, University Medical Center Groningen, and Groningen Research Institute for Asthma and COPD, Groningen, the Netherlands; 3 Pulmonology Department, Erasmus Medical Centre, Rotterdam, the Netherlands; 4 Department of Epidemiology, Medisch Spectrum Twente, Enschede, The Netherlands; 5 Department of Pulmonary Medicine, Catharina Hospital, Eindhoven, The Netherlands; 6 Department of Pulmonology, Northwest Clinics, Alkmaar, the Netherlands; 7 Department of Primary and Community Care, Radboud University Medical Center, Nijmegen, The Netherlands; 8 Department of Clinical Pharmacy & Pharmacology Groningen Research Institute for Asthma and COPD (GRIAC), University Medical Center Groningen, University of Groningen, Groningen, The Netherlands; 9 Department of Pulmonary Medicine, Spaarne Gasthuis, Haarlem, The Netherlands; 10 Department of Pulmonary Medicine, Albert Schweitzer Ziekenhuis, Dordrecht, The Netherlands; 11 Department of Pulmonary Medicine, HagaZiekenhuis, The Hague, The Netherlands; 12 Department of Pulmonary Medicine, Gelre Ziekenhuizen, Zutphen, The Netherlands; 13 Department of Respiratory Medicine, Van Weel-Bethesda Hospital, Dirksland, The Netherlands; 14 Department of Respiratory Medicine, Medical Centre Leeuwarden, Leeuwarden, The Netherlands; 15 Department of Pulmonology, Haaglanden Medical Centre, The Hague, The Netherlands; 16 Department of Clinical Pharmacy, Franciscus Gasthuis, Rotterdam, The Netherlands

**Keywords:** COPD, Adherence, Triple therapy, Single inhaler, Smart inhaler, Telemonitoring, eHealth, Digital adherence technology

## Abstract

**Background:**

Medication non-adherence is a significant problem in patients with Chronic Obstructive Pulmonary Disease (COPD). Efforts to address this issue are receiving increased attention. Simplifying treatment by prescribing single-inhaler triple therapy (SITT) as an alternative to multi-inhaler triple therapy (MITT) or with smart inhalers are often considered potential solutions. However, the actual impact of these innovations on adherence and clinical outcomes is unclear.

**Methods:**

To address this knowledge gap we first conducted a literature review focusing on two research questions: 1) the difference in adherence between SITT and MITT users in COPD, and 2) the effect of smart inhalers on adherence in COPD. Separate searches were conducted in PubMed and two authors independently assessed the articles. In addition, we present a protocol for a study to acquire knowledge for the gaps identified.

**Results:**

To address the first research question, 8 trials were selected for further review. All trials were observational, i.e. randomized controlled trials were lacking. Seven of these trials showed higher adherence and/or persistence in patients on SITT compared with patients on MITT. In addition, four studies showed a positive effect of SITT on various clinical outcomes. For the second research question, 11 trials were selected for review. While most of the studies showed a positive effect of smart inhalers on adherence, there was considerable variation in the results regarding their effect on other clinical outcomes.

The TRICOLON (TRIple therapy COnvenience by the use of one or multipLe Inhalers and digital support in ChrONic Obstructive Pulmonary Disease) trial aims to improve understanding regarding the effectiveness of SITT and smart inhalers in enhancing adherence. This open-label, randomized, multi-center study will enroll COPD patients requiring triple therapy at ten participating hospitals. In total, 300 patients will be randomized into three groups: 1) MITT; 2) SITT; 3) SITT with digital support through a smart inhaler and an e-health platform. The follow-up period will be one year, during which three methods of measuring adherence will be used: smart inhaler data, self-reported data using the Test of Adherence to Inhalers (TAI) questionnaire, and drug analysis in scalp hair samples. Finally, differences in clinical outcomes between the study groups will be compared.

**Discussion:**

Our review suggests promising results concerning the effect of SITT, as opposed to MITT, and smart inhalers on adherence. However, the quality of evidence is limited due to the absence of randomized controlled trials and/or the short duration of follow-up in many studies. Moreover, its impact on clinical outcomes shows considerable variation. The TRICOLON trial aims to provide solid data on these frequently mentioned solutions to non-adherence in COPD. Collecting data in a well-designed randomized controlled trial is challenging, but the design of this trial addresses both the usefulness of SITT and smart inhalers while ensuring minimal interference in participants' daily lives.

**Trial registration:**

NCT05495698 (Clinicaltrials.gov), registered at 08–08-2022. Protocol version: version 5, date 27–02-2023.

**Supplementary Information:**

The online version contains supplementary material available at 10.1186/s12890-024-03044-3.

## Introduction

### Background

Chronic obstructive pulmonary disease (COPD) is characterized by chronic respiratory symptoms due to abnormalities in the airways and/or alveoli, resulting in persistent and often progressive airflow obstruction. Inhalation medication is the primary medical treatment, with three types of inhalation medication available as maintenance therapy: 1) long-acting β2-agonist (LABA), 2) long-acting muscarinic antagonist (LAMA), and 3) inhaled corticosteroids (ICS). Triple therapy is defined as treatment with LABA, LAMA and ICS [1]. Large randomized controlled trials have shown that triple therapy provides clinical benefits compared to dual therapy in patients with moderate-to-severe COPD and a history of exacerbations, particularly when eosinophilia is present [2–6]. Furthermore, two trials suggest that triple therapy reduces mortality in this specific population [7, 8]. Triple therapy can be administered through multiple devices, known as multi-inhaler-triple therapy (MITT), or combined in one inhaler, known as single-inhaler-triple therapy (SITT). Assessment of adherence to inhalation therapy is a crucial element in managing COPD patients according to the Global Initiative for Chronic Obstructive Lung Disease (GOLD) [1]. Previous studies have shown that medication adherence is poor in patients with COPD. A systematic review showed that non-adherence rates ranged from 22 to 93%, depending on the study population and method of measurement [9]. Non-adherence is associated with poor clinical and economic outcomes [10, 11]. Simplifying treatment with smart-inhalers and prescribing single-inhaler triple therapy (SITT) as an alternative to multi-inhaler triple therapy (MITT) can be considered as potential solutions. Nowadays, the GOLD report (version 2024) acknowledges that a single inhaler may be more convenient compared to multi-inhaler therapy. [1]. The actual impact of these innovations on adherence is unclear. Therefore, we conducted a literature review to examine the current evidence on these two potential solutions. Should the evidence prove to be insufficient, we wanted to present a protocol for a study to fill this gap.

### Methods for measuring adherence

Adherence is defined as the process by which patients take their medication as prescribed, while persistence refers to the duration from initiation to discontinuation of the treatment. Adherence and persistence are complex constructs, as previously described by Vrijens et al. [12, 13]. Various methods can be used to assess medication adherence. Healthcare professionals commonly inquire directly about their patients' adherence. While this approach is straightforward, research has demonstrated its unreliability in comparison to more objective measurement methods. Unstructured self-reports often lead to an overestimation of adherence [14]. Patients can structurally self-report their adherence using questionnaires, such as the Test of Adherence to Inhalers (TAI). The TAI is developed specifically to measure adherence to inhalation medication in patients with COPD or asthma [15]. Furthermore, pharmacy data are often used to determine patient’s access to medication over time by calculating the Proportion of Days Covered (PDC). This method may be less reliable due to missing or inaccessible data (e.g. when multiple pharmacies are used) and the uncertainty about whether the patient actually used the medication [16, 17]. Smart inhalers offer a more objective method for measuring adherence. These electronic sensors (e-devices) are attached to or integrated into inhalers. Devices range from simple dose counters to advanced devices that provide reminders, feedback, and/or analyse inhalation technique [18]. Smart inhalers are often integrated with other e-health interventions, such as telemonitoring, personalised feedback with apps, counselling, and training [19–21]. Finally, hair analysis can provide a bioanalytical assessment of average long-term drug exposure in the human body. This method could potentially provide an objective measure of adherence over the last few months. However, external factors can impact measurement, and only small part of medication that has been present in the systemic blood circulation is built into hair [22].

## Methods

First, we present a review of the literature on two strategies to improve adherence. Second, we describe the study protocol of the TRICOLON trial (TRIple therapy COnvenience by the use of one or multipLe Inhalers and digital support in ChrONic Obstructive Pulmonary Disease).

### Search strategy literature review

Separate searches were conducted in PubMed based on two research questions: 1) What is the difference in adherence between SITT and MITT users in COPD?, and 2) What is the effect of smart inhalers on adherence in COPD? The selection of articles from the second PubMed search was supplemented by four papers known to the authors or found by snowballing. Two authors independently assessed these articles to determine whether they should be included in this review (LC and JiV). In cases of disagreement, the opinion of a third author was sought (HK). Only original English-language studies were included. Details of the PubMed search and selection process can be found in the Appendix, Suppl.1.

### Assessment of the evidence

The primary outcome was the adherence and/or persistence to triple therapy. Secondary outcomes were clinical outcomes, such as exacerbations, COPD Assessment Test (CAT) score, and FEV_1_. Each study underwent an evaluation across multiple criteria to assess the evidence supporting the research question. The strength of the study design was rated with stars: one star for retrospective studies, two stars for prospective observational studies or intervention studies without randomization (e.g. before-and-after designs), and three stars for randomised controlled trials. A green smiley indicates statistically significant superior results in the intervention group (either SITT or smart-inhaler group, depending on the research question) compared to the control group; a yellow neutral smiley signifies no difference between the groups, while a red sad smiley denotes statistically significant inferior results in the intervention group compared to the control group. Additionally, a thumbs-up signifies that other clinical outcomes were measured in that study, whereas a thumbs-down indicates the absence of measurements for other clinical outcomes, Tables 1 and 2.

**Table 1:**
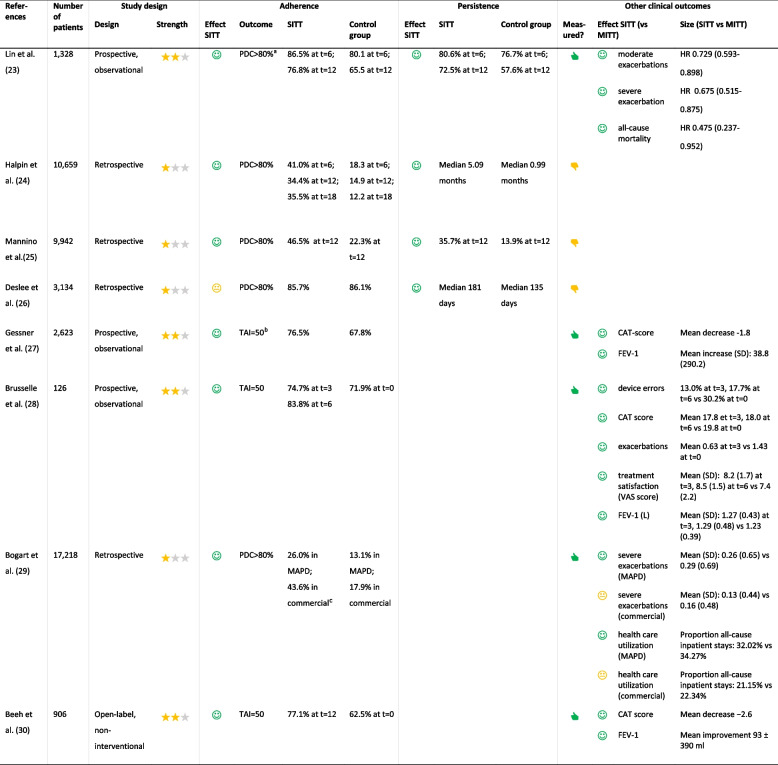
Overview of trials investigating adherence to single-inhaler triple therapy vs multi-inhaler triple therapy in COPD patients

Evaluation of the evidence. Strength of the study design:  = retrospective,  = prospective observational or intervention study without randomization, and  = randomised controlled trial. Effect SITT on outcome (vs MITT):  = statistically significant better result in SITT compared to MITT,  = no statistically significant difference between SITT and MITT, and  = statistically significant worse result in SITT compared to MITT. Other clinical outcomes measured:  = yes,  = no

^a^*PDC *Proportion of days covered. PDC > 80% defines good adherence

^b^*TAI *Test of Adherence to Inhalers. TAI = 50 defines good adherence

^c^ Results are presented separately for patients enrolled in either Medicare Advantage with Part D (MAPD) or commercial insurance [29]

**Table 2:**
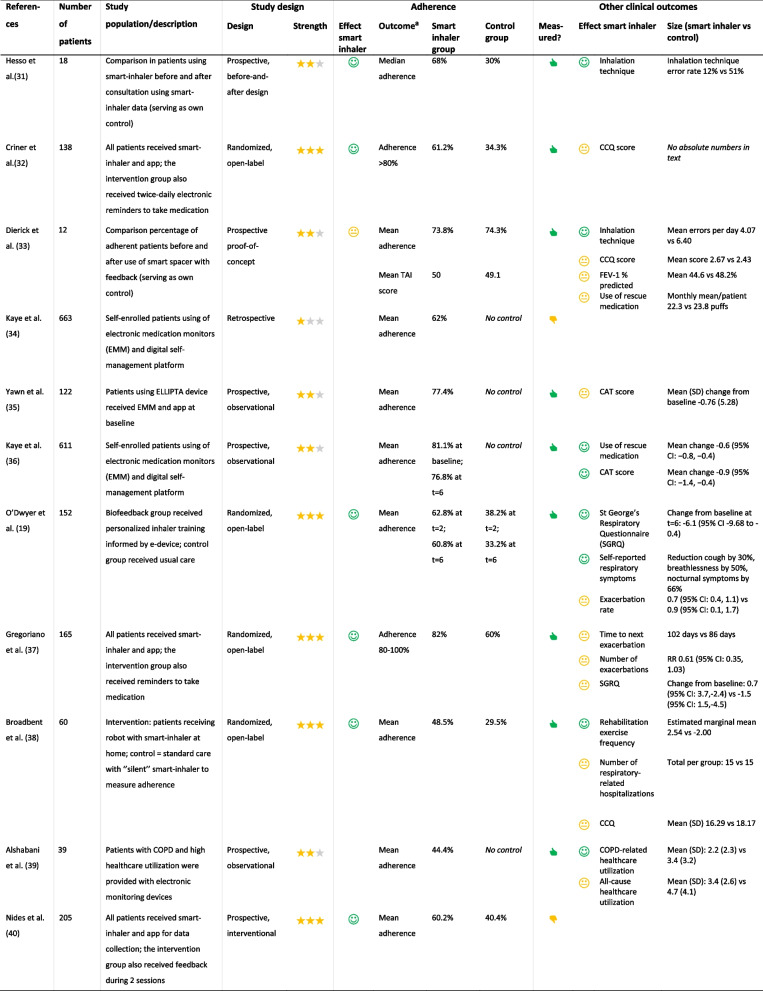
Overview of trials investigating the effect of smart-inhalers on adherence in COPD patients

Evaluation of the evidence. Strength of the study design:  = retrospective,  = prospective observational or intervention studies without randomization, and  = randomised controlled trial. Effect smart inhaler on adherence (vs control group without smart inhaler):  = statistically significant better result in intervention group compared to controls,  = no statistically significant difference between intervention group compared to controls, and  = statistically significant worse result in intervention group compared to controls. Other clinical outcomes measured:  yes,  no

^a^measured using smart inhaler unless specified otherwise [19, 31–40]

## Results

### Literature review

#### Adherence to single versus multi inhaler triple therapy

The first search yielded eighteen articles, Suppl. Figure 1. After the selection process, eight articles were included for further evaluation, Table 1. None had a randomized controlled design. Four studies compared SITT with MITT in a prospective setting [23, 27, 28, 30] and four in a retrospective analysis [24–26, 29]. The retrospective studies used either anonymized data from electronic health records of patients in primary or secondary care [24, 26], or databases of health insurance claims [25, 29]. Seven of these studies showed that adherence and/or persistence to SITT was significantly higher compared to MITT in COPD patients [23–25, 27–30]. Deslee et al. presented contrasting findings, showing that while persistence was higher in the SITT group (median 181 versus 135 days), adherence levels were similar in SITT and MITT (85.7% versus 86.1%) [26]. The seven trials that showed a positive effect of SITT on adherence were heterogeneous in terms of study design, and methods of measurement, type of inhaler, and molecules. Although most studies directly compared SITT with MITT, two of the prospective studies assessed adherence to SITT after switching from either dual therapy or MITT [27, 28]. Brusselle et al. reported separate results for former MITT users: 71.9% at baseline, 74.7% three months after switching to SITT, and 83.8% at six months [28]. In contrast, Gessner et al. did not report separate results for the different treatments at baseline. Therefore, this difference between SITT and the control group should be interpreted with caution [27].

#### Clinical outcomes in single vs multi inhaler triple therapy

Five trials investigated the effect of SITT compared to MITT on clinical outcomes. Four of these trials showed a beneficial effect of SITT on clinical outcomes, Table 1. SITT users had a lower risk of exacerbations compared to MITT users in two studies [23, 28]; three studies showed lower CAT-scores and higher FEV_1_ in SITT users [27, 28, 30]; and one study showed a reduced all-cause mortality risk in SITT users [HR: 0.475 (0.237–0.952), *p* = 0.036] [23]. In contrast to these studies with positive effects of SITT, Bogart et al. showed different results in two different groups of patients. In patients enrolled in Medicare Advantage with Part D (MAPD) insurance, a significant reduction in exacerbations and healthcare utilisation was seen in the SITT group. However, these differences were not statistically significant in the commercially insured patients [29].

#### The effect of smart inhalers on adherence

The second part of our literature review investigated the effect of smart inhalers on adherence in patients with COPD and resulted in 78 articles, from which 11 studies were selected, Table 2. These studies were heterogenetic in terms of number of patients, study design, and type of intervention and/or smart inhaler. Additionally, length of follow-up ranged from 1 to 12 months, with the vast majority (90.9%) having a follow-up period of ≤ 6 months. Seven studies prospectively compared COPD patients who used a smart-inhalers with a control group; four of those had a randomized controlled open-label design. Six of these seven studies showed a statistically better adherence in the intervention group [19, 31, 32, 37, 38, 40]; one prospective study showed no difference in adherence between smart inhaler users and controls [33]. Four other observational studies showed variable average adherence rates in smart-inhaler users from 44–77%; however, it should be noted that these trials did not include a control group without smart-inhaler [34–36, 39].

#### The effect of smart inhalers on clinical outcomes

Nine studies also investigated the effect of smart inhalers on clinical outcomes [19, 31–33, 35–39]. These studies showed mixed results, Table 2. A significant better inhalation technique was seen in two studies [31, 33]. The impact of smart inhalers on other clinical outcomes, including exacerbations, disease burden, quality of life assessments, rescue medication usage, hospitalizations, and healthcare utilization, varied across different studies [19, 32, 33, 35–39].

#### Conclusion of literature review

This review suggests promising results concerning the effect of SITT, as opposed to MITT, and smart inhalers on adherence. However, the quality of evidence is limited due to the absence of randomized controlled trials and/or the short duration of follow-up in many studies. Moreover, there is considerable variation in the findings of these studies regarding diverse clinical outcomes. Consequently, there is a clear need for comprehensive randomised controlled trials to evaluate the benefits of SITT versus MITT in COPD, as well as the individual and combined effects of smart inhalers, both on adherence and clinical outcomes.

### Study protocol for an RCT: the TRICOLON study

The TRICOLON study was initiated to provide evidence on two previously mentioned potential solutions for the non-adherence issue in COPD patients. The primary objective is to investigate whether the adherence to SITT is superior to the adherence to MITT over 12 months of treatment and to investigate whether the adherence of SITT users with a smart inhaler and digital support is superior to the adherence of MITT and SITT users without the smart inhaler and digital support. As a secondary objective, three methods of measuring adherence will be compared: smart inhaler data, self-reported data using the Test of Adherence to Inhalers (TAI) questionnaire [15], and drug analysis of formoterol in scalp hair samples [41]. Finally, differences in clinical outcomes between the study groups will be examined.

#### Study design

**Fig. 1:**
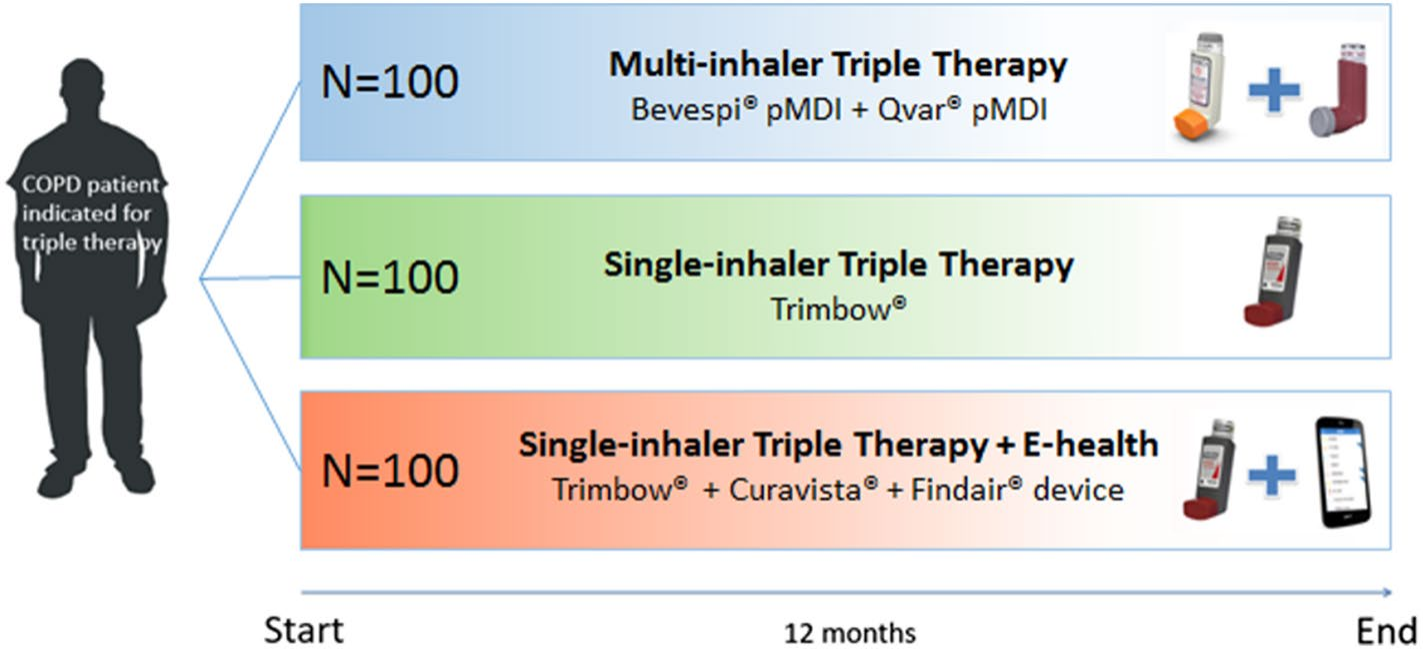
Study design. Patients are randomly assigned in a 1:1:1 ratio to one of three groups: 1) multi-inhaler triple therapy (Bevespi® and Qvar®), 2) single-inhaler triple therapy (Trimbow®), 3) single-inhaler triple therapy (Trimbow®) with e-health. The follow-up period is one year

**Fig. 2:**
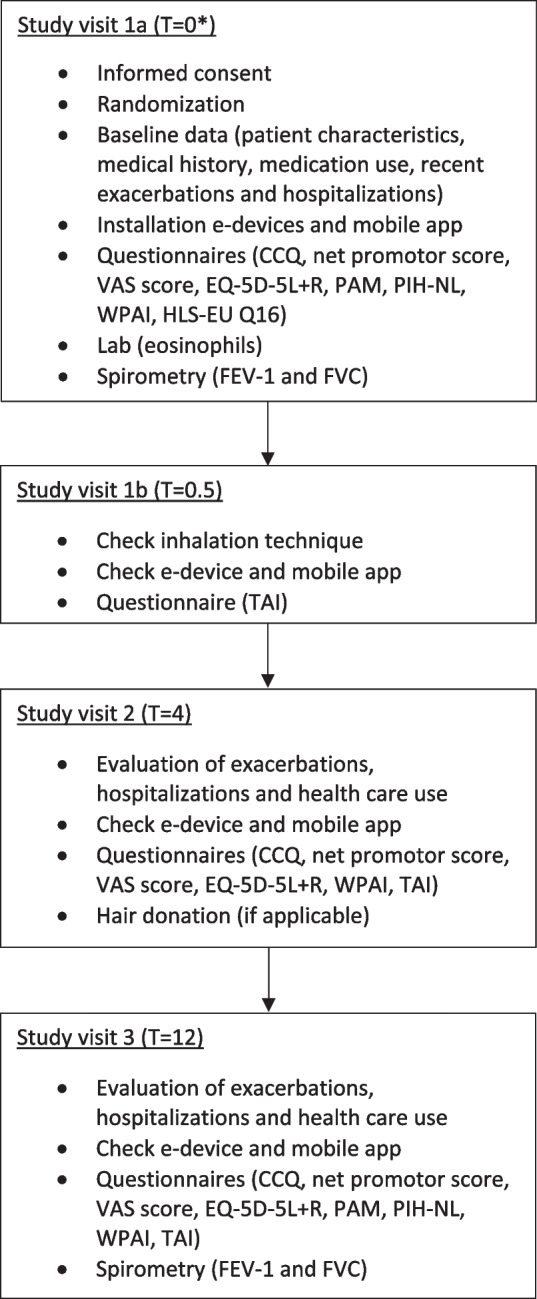
Study program *time in months

The TRICOLON study is an investigator-initiated, prospective, open-label, randomised, real-world, multicentre study. The study will be conducted at ten hospitals in the Netherlands, Supplement 2. Patients will be recruited from the pulmonary wards or outpatient clinics of the participating hospitals. Informed consent will be obtained by a member of the research team. Participants are randomly assigned in a 1:1:1 ratio to one of three groups: 1) multi-inhaler triple therapy (Bevespi® and Qvar®), 2) single-inhaler triple therapy (Trimbow®), 3) single-inhaler triple therapy (Trimbow®) with digital support, Fig. 1. The follow-up period will be one year, during which we aim to minimise disruption to their usual care, thus creating a (close to) real-world situation. The study outline is presented in Fig. 2. Ethical approval for this study was granted by the United Medical Research Ethics Committees (NL79938.100.22). The trial is registered on clinicaltrials.gov (NCT05495698).

#### Study participants

A total of 300 patients will be enrolled. Patients with a diagnosis of COPD and an indication for triple therapy according to their physician following the GOLD report are eligible to participate. Patients are excluded if asthma is the dominant diagnosis (asthma in the past or as a comorbidity is allowed), if patients use nebulizers or if they already use an e-health application for their COPD. The inclusion and exclusion criteria are displayed in Table 3.

**Table 3:** In- and exclusion criteria

Inclusion criteria
• Clinical diagnosis of COPD for at least 1 year before the screening visit • Aged 40 years and older • An indication for triple therapy according to the treating physician^a^ • Owner of mobile device compatible with e-device app with access to internet • Current or ex-smoker • Willing to provide written informed consent
**Exclusion criteria**
• Asthma as the predominant disease according to the investigator’s opinion; comorbidity or a past history of asthma is allowed • Use of e-health application for COPD in the past six months • Use of nebulized bronchodilators • Insufficiently skilled in the Dutch language to be able to read and understand the app. Help by third party (family members) is allowed • Inability to comply with study procedures or with study treatment • Pregnant or lactating women and all women physiologically capable of becoming pregnant unless they have highly effective contraceptive

^a^Could be step-up from dual therapy or currently receiving triple therapy (both MITT and SITT)

#### Interventions

The study groups have uniform doses, identical molecules and doses (beclomethasone 100µg, formoterol 6µg, and glycopyrronium 10µg), and the same device type (pressurised metered dose inhaler). Therefore, the only differentiating factors are the number of devices used and the use of digital support. Digital support comprises the Findair® smart-inhaler, an electronic device that is attached to the inhaler and measures the frequency and time of the actuations, and the Curavista® platform (Gezondheidsmeter PGO), a digital platform that promotes patient self-management by displaying their medication use and providing medication intake reminders [31, 32]. Each patient is provided with smart inhalers that are attached to all their inhalers, including the potential rescue medication. This enables the measurement of actuation frequency and timing. The smart-inhalers are linked to the digital platform for data collection. However, it is important to note that only group 3 has access to an overview of the actuations with feedback in the app and reminders, along with full access to the digital platform. Groups 1 and 2 will not receive any reminders and cannot access the digital platform. Their e-health applications are solely used for data collection purposes, ensuring "silent monitoring" that should not influence their adherence.

We aim for all participants to complete the study using the original study medication. However, given the real-world nature of the study, it is possible that patients require a change in medication due to clinical reasons such as side effects or lack of effectiveness. To prevent the loss of these patients from the study, changes in medication are permitted only when absolutely necessary. Data collection will persist via the electronic devices and with the same settings of the app (silent monitoring or full access). Patients who no longer use a pressurized metered dose inhaler will be excluded, as the electronic devices are specifically tailored for this type of inhaler.

#### Outcomes

The primary outcome is the average adherence to ICS therapy (measured as the number of actuations registered by the smart-inhaler divided by the total number of doses prescribed, in %) over 12 months of treatment. As a secondary outcome, the average adherence to LABA/LAMA in study group 1 will be compared with adherence to ICS in group 1, and with adherence to LABA/LAMA/ICS in groups 2 and 3. Additional secondary outcomes include the percentage of patients with good adherence, defined as an average ICS adherence of more than 80% and less than 110% actuations measured by the smart inhaler; the Test of Adherence to Inhalers (TAI) scores; and drug levels of formoterol in scalp hair. The hair samples will be collected, stored, and prepared according to the guidelines of the Society of Hair Testing [42]. Additionally, the study will measure changes in Patient Reported Outcome Measures (PROMs), use of rescue medication, number of exacerbations and hospitalizations, healthcare consumption, and spirometry (FEV_1_). The PROMs are displayed in Supplement 3.

#### Statistical analysis

The statistical software G*Power version 3.1.9.6 was used to calculate the sample size. The study aims to compare three groups: group 1 vs group 2, group 2 vs group 3, and group 1 vs group 3. The sample size calculation is based on the comparison of the average adherence in group 1 vs group 2 (so MITT vs SITT without e-health). Based on a previous study [19], we expect a 15% difference between the groups, with a standard deviation of 30%. The significance level of the test was set at (alpha) 0.0167 using the Holm-Bonferroni method to adjust for multiple testing, as we are comparing three groups (alpha 0.05/3 = 0.0167), with a power of 80%. We calculated n = 84 for each group using a two-sided T-test, and plan to include 100 patients in each group of the study, considering a potential drop-out rate of 15%. The patients will be randomized using the program Castor EDC. To prevent inequality in the study groups, at randomization patients will be stratified for their treatment before the study (dual therapy, MITT or SITT) and for inclusion during an exacerbation or during stable disease. The data will be analyzed using both the intention-to-treat (primary analysis) and per-protocol methods. The Kruskal–Wallis test or ANOVA, as appropriate, will be used to compare the average adherence between the three study groups. Post-hoc comparisons will be performed using Mann–Whitney U tests or Tukey’s HSD, as appropriate. The average baseline scores of the PROMs and baseline clinical status (FEV_1_, number of exacerbations, hospitalizations and rescue medication use) will be presented descriptively. These scores and outcomes are all continuous, unpaired data and will be compared between more than two groups. Therefore, we will use the Kruskal–Wallis or ANOVA, as appropriate, to investigate the differences between the three groups. The Net Promoter score is a binary variable, so we will use the Chi-square test. Questionnaire data will be analysed by both the difference in mean scores between groups and the percentage of patients achieving the minimally clinical important difference (MCID) when available. Furthermore, a mixed model repeated measurement analysis will be performed to assess the differences in the previously mentioned outcomes over time. The analysis will be conducted on normally distributed data, with or without transformations. The main parameter of interest is the group * time interaction. Between-group comparisons will be adjusted using the method of Sidak. If the data cannot be normalized, difference scores will be calculated (follow-up – baseline). These differences will be analysed using either the Kruskal–Wallis test or ANOVA, where appropriate. Post-hoc comparisons will be conducted using Mann–Whitney U tests or Tukey’s HSD, where appropriate.

#### Safety

Serious adverse events (SAEs) will be monitored and reported in accordance with the legal requirements and deadlines. The Ethics committee has granted permission for hospital admissions resulting from a COPD exacerbation not to be reported as SAE. This exemption is due to their frequent occurrence in this specific population. Instead, to monitor this fragile population, a Data and Safety Monitoring Board (DSMB) has been established. The DSMB will periodically review mortality rates, serious adverse events, and premature withdrawals from the study every six months. The application and e-health platform in use have a substantial history, spanning many years. Over the past two decades, 26 peer-reviewed scientific publications have been published. Notably, there have been no reported safety concerns, and assessments of usability, feasibility, and efficacy consistently yield positive results [43].

## Discussion

Non-adherence is a significant challenge in COPD patients, and addressing this issue is receiving increased attention. The first solution we investigated was simplifying the treatment by prescribing single-inhaler triple therapy (SITT) as an alternative to multi-inhaler triple therapy (MITT). The GOLD report 2023 suggested, for the first time, that single inhaler therapy may be more convenient than multi inhaler therapy [1]. However, although our literature review reveals some promising results regarding the effect of SITT on adherence, the quality of evidence is limited due to the absence of randomized controlled trials that specifically examined the difference in adherence between SITT and MITT. Due to the observational setting and design of all studies in the first part of this review, the SITT and MITT groups showed differences in their baseline characteristics, including the number of exacerbations prior to enrolment, disease severity, and FEV-1. Moreover, the quality of the observational data was occasionally limited. For example, two studies relied on administrative databases using health insurance claims [25, 29]. Consequently, from these trials we are unable to draw conclusions on the cause-effect relationship between improved adherence and clinical outcomes. In contrast to the four studies included in our review, which all demonstrated slightly better clinical outcomes in SITT users, other literature, not incorporated into our review due to a lack of adherence as an outcome, presented contrasting results regarding the effect of SITT on clinical outcomes. Specifically, a retrospective study in Spain and an RCT showed improved clinical outcomes in SITT users [44, 45]. However, three randomized controlled trials indicated that both SITT and MITT users exhibited similar results in terms of lung function, health status, exacerbation rate, and rescue medication usage [2, 46, 47].

The use of smart inhalers was the second potential solution to non-adherence that we investigated. Our review showed that while the effect on adherence was mostly positive, no consistent differences in clinical outcomes were observed. The strength of studies showing improved adherence in the absence of improved clinical outcomes is limited. These findings are consistent with other recent reviews in both COPD and asthma [18, 20, 21]. Demonstrating the connection between adherence and clinical outcomes has proven to be challenging. Given that COPD entails irreversible lung damage and the medication aims to stabilize rather than cure the disease, an extensive follow-up period is essential to demonstrate its impact on clinical outcomes. However, the current studies have limitations regarding the duration of follow-up. The smart inhalers used, as well as the supplementary interventions, and the extent of monitoring and/or interference varied significantly among the studies. The majority of smart inhalers used in current literature can only record the time and location of actuation. They are unable to measure whether inhalation is performed correctly. Other techniques, such as smart inhalers capable of measuring airflow or hair analysis of inhaled drugs, can assist in addressing this limitation. Further research is needed to gain a deeper understanding of the contributions of the different electronic modalities, the underlying mechanisms, clinical outcomes, and optimal implementation of these devices in clinical practice. Several challenges must be addressed before integrating smart inhalers into daily practice, including technical complexities, limited evidence concerning clinical outcomes, uncertainties about cost-effectiveness, and the issue of funding for the devices [18]. The TRICOLON study aims to offer additional evidence, potentially bringing us closer to their use in daily practice.

To the best of our knowledge, the TRICOLON trial is the first that aims to investigate whether single inhaler usage in COPD patients receiving triple therapy can improve adherence in a large-scale, randomized, controlled, real-world setting. Moreover, unlike the majority of existing studies focusing on the impact of smart inhalers, the TRICOLON trial distinguishes itself with a prolonged follow-up period of one year. We acknowledge that patients’ adherence may be influenced by their awareness of participating in a study. To minimize this interference, we have limited the number of study visits to closely resemble real-world settings. Additionally, patients are informed that the focus of this study is on the convenience of various treatment options, and adherence is not specifically mentioned in the information provided by the researchers or in the patient information letter. This has been approved by the Medical Ethical Review Board. The Tricolon study not only investigates the impacts of a single inhaler and the use of a smart inhaler on adherence but also evaluates various clinical outcomes, including exacerbation rates, hospitalizations, and disease burden. Nevertheless, the study is powered on the primary outcome adherence to inhalation therapy. Therefore, although not powered for the clinical outcomes or for the correlation between adherence and clinical outcomes, valuable information on these outcomes will be collected to inform possible follow-up studies.

The implementation of an e-health application may present challenges, whether due to technical issues or the potential unfamiliarity among patients and healthcare professionals [48]. To proactively address these concerns, we dedicated time to thoroughly test the application before the start of the study. This involved multiple stakeholders, including the researchers, the app producer, and patients. Moreover, the application's design is intentionally kept simple and clear, ensuring that individuals of all ages, educational backgrounds, and health literacy levels can easily comprehend and use it. This was confirmed in a previous study where an older, and thus more digitally challenged, population of patients with Idiopathic Pulmonary Fibrosis (IPF) used this app for daily spirometry. The study found that 80% of the participants found the app easy to use, and 90% did not perceive it as burdensome [49].

Beyond addressing the primary research questions, this TRICOLON study creates an opportunity for a direct comparison between three methods to measure adherence: digital data from the smart inhaler, self-reported data collected through the TAI questionnaire, and drug deposition data in hair. The use of this final technique is relatively uncommon in studies concerning inhalation medication, although it has been used in previous research related to cortisol and in forensic studies [22, 50]. In a specific study involving asthma and COPD patients, the measurement of inhalation medication demonstrated a clear dose–response relationship among those using formoterol [41].

Our identification of a lack of high-quality data on the improvement of adherence of SITT over MITT therapy in COPD and limited data for smart inhalers highlights the need for further research. The multi-centre, randomized controlled, three-arm, real-world TRICOLON trial aims to increase insight in the value of SITT and the added value of electronic adherence monitoring.

### Supplementary Information

Suuplementary Material 1.

## Data Availability

All data relevant to the study are included in the article or uploaded as supplementary information. The datasets used and/or analyzed during the current study are available from the corresponding author on reasonable request.
