# Supplementary material for: Adherence to single inhaler triple therapy and digital inhalers in Chronic Obstructive Pulmonary Disease: a literature review and protocol for a randomized controlled trial (TRICOLON study)
Source: BMC Pulm Med. 2024 Jul 4;24:317. doi: 10.1186/s12890-024-03044-3 (PMC11225120; doi:10.1186/s12890-024-03044-3)
Supplement: Supplementary file 1 — Suuplementary Material 1. [file 12890_2024_3044_MOESM1_ESM.docx]

# Appendix

**Supplement 1. Article selection for literature review**

Research question 1: what is the difference in adherence between SITT vs MITT users in COPD?

P: patients with COPD

I: single-inhaler triple therapy users

C: multi-inhaler triple therapy users

O: adherence

Type of study design: original papers

Search date: 19-01-2024

Search Strategy for literature review ‘Adherence in SITT vs MITT users’ in PubMed

("Lung Diseases, Obstructive"[Mesh] OR obstructive-lung-disease*[tiab] OR COPD[tiab] OR Obstructive-pulmonary-disease*[tiab] OR COAD[tiab] OR obstructive-airway-disease*[tiab] OR chronic-airflow-obstruction*[tiab] OR chronic-airway-obstruction*[tiab] OR bronchitis*[tiab] OR bronchiolit*[tiab] OR emphysem*[tiab] OR bronchopneumoni*[tiab] OR broncho-pneumoni*[tiab] OR cryptogenic-organizing-pneumoni*[tiab] OR BOOP[tiab]) AND (single-inhaler*[tiab] OR (inhal*[tiab] AND (single-device*[tiab] OR single-aerosol*[tiab]))) AND (multi-inhaler*[tiab] OR multiple-inhaler*[tiab] OR (inhal*[tiab] AND (multi-device*[tiab] OR multiple-device*[tiab] OR multiple-aerosol*[tiab]))) AND ("Treatment Adherence and Compliance"[Mesh] OR adher*[tiab] OR comply*[tiab] OR complian*[tiab]) AND (English[lang] OR Dutch[lang])

**Suppl. Figure 1**

**
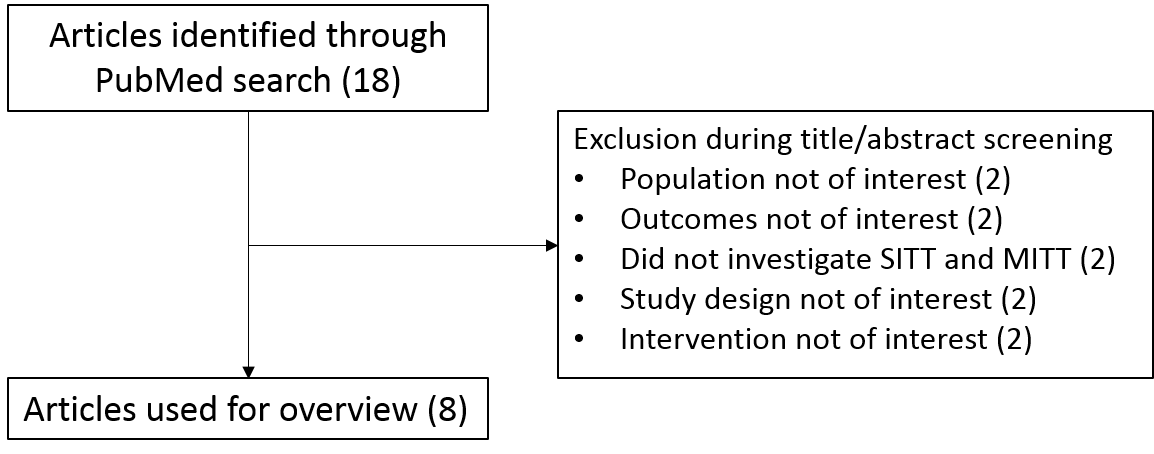
**

Research question 2: what is the effect of smart-inhalers on the adherence in COPD?

P: patients with COPD

I: smart-inhaler

C: usual care without smart-inhaler

O: adherence

Type of study design: orginal papers

Search date: 19-01-2024

Search Strategy for literature review ‘Effect of smart-inhaler on adherence’ in PubMed

("Pulmonary Disease, Chronic Obstructive"[Mesh] OR COPD[tiab] OR chronic-obstructive-pulmonary-disease*[tiab] OR COAD[tiab] OR chronic-obstructive-airway-disease*[tiab] OR chronic-airflow-obstruction*[tiab] OR chronic-airway-obstruction*[tiab]) AND ("Digital Technology"[Mesh] OR "Robotics"[Mesh] OR "Reminder Systems"[Mesh] OR smart-inhaler*[tiab] OR smart-device*[tiab] OR electronic-device*[tiab] OR e-device*[tiab] OR electronic-monitoring-device*[tiab] OR electronic-medication-monitor*[tiab] OR digital-inhaler*[tiab] OR digital-device*[tiab] OR digital-technolog*[tiab] OR electronic-inhaler*[tiab] OR digihaler*[tiab] OR digital-health-tool*[tiab] OR digital-health-device*[tiab] OR interactive-monitor*[tiab] OR robot*[tiab] OR e-health-application*[tiab] OR ehealth-application*[tiab] OR health-technolog*[tiab] OR ehealth-technolog*[tiab]) AND ("Treatment Adherence and Compliance"[Mesh] OR "Patient Compliance"[Mesh] OR adher*[tiab] OR nonadher*[tiab] OR comply*[tiab] OR noncomply*[tiab] OR complian*[tiab] OR noncomplian*[tiab]) AND (English[lang] OR Dutch[lang])

**Suppl. Figure 2**


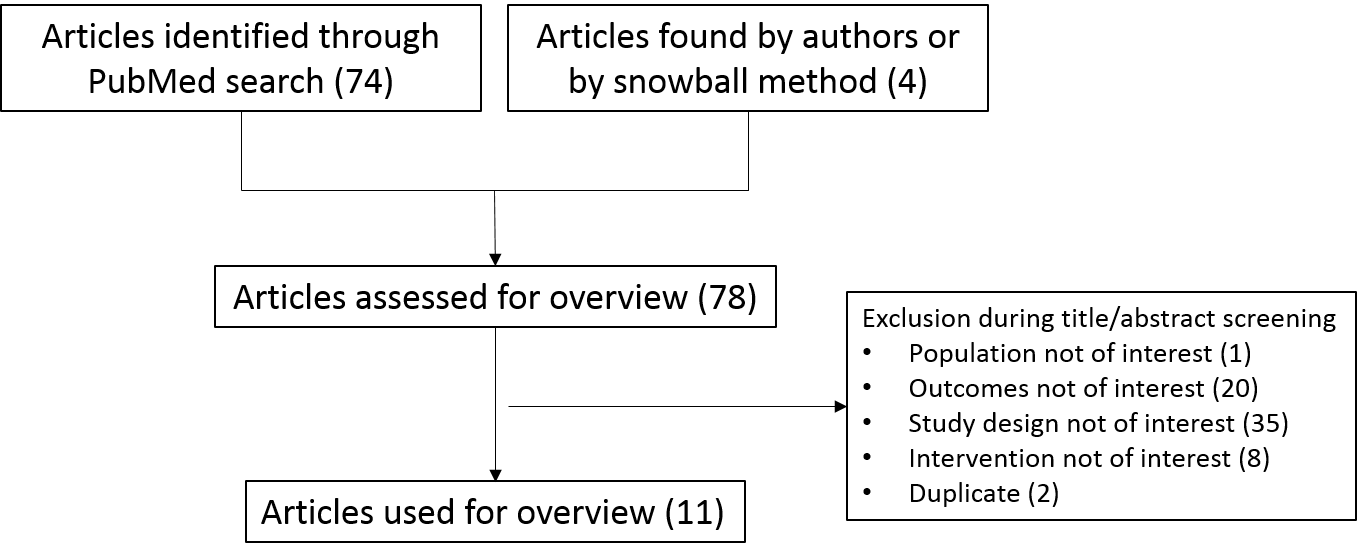


**Supplement 2. Participating hospitals Tricolon trial**

1. Franciscus Gasthuis & Vlietland Hospital, Rotterdam, The Netherlands (coordinating center)
2. Spaarne Gasthuis, Haarlem, The Netherlands
3. Northwest Clinics, Alkmaar, the Netherlands
4. Medical Centre Leeuwarden, Leeuwarden, the Netherlands
5. Albert Schweitzer ziekenhuis, Dordrecht, The Netherlands
6. Van Weel-Bethesda Hospital, Dirksland, the Netherlands
7. Gelre Ziekenhuizen, Zutphen, The Netherlands
8. Haaglanden Medical Centre, The Hague, the Netherlands
9. Catharina Hospital, Eindhoven, The Netherlands
10. HagaZiekenhuis, The Hague, The Netherlands

**Supplement 3. Patient Reported Outcome Measures (PROMs) of the TRICOLON trial**

*Clinical COPD Questionnaire (CCQ)*: 10-item questionnaire about symptom severity in the past seven days and health-related quality of life. Total score ranges from 0 to 6, where a higher score indicates a worse health status(48).

*Net promotor score*: contains one question: how likely is it that you would recommend this treatment to a friend or family? Scale ranges from 0 (not likely) to 10 (very likely)(49).

*VAS score*: a non-specific scale to score patient’s satisfaction about his/her treatment, ranging from 0 (not satisfied at all) to 10 (extremely satisfied).

*The 5-level EQ-5D version with respiratory bolt-on (EQ-5D-5L+R)*: questionnaire to assess the health-status on different domains (mobility, self-care, daily activities, pain/discomfort and fear/depression). A sixth question about respiratory problems was added to the EQ-5D-5L (50).

*Patient Activation Measure (PAM)*: an assessment of patients’ knowledge, skills and confidence for managing their own health status. The questionnaire has a 0–100 scale and gives insight in the activation level and health-related characteristics as attitudes, motivators, and behaviours(51).

*Partners in Health Scale- the Netherlands (PIH-NL)*: 12-item questionnaire to assess self-management behaviour and knowledge of patients with chronic diseases(52).

*Work Productivity and Activity Impairment (WPAI)*: questionnaire to measure impairments in work and activities(53).

*16-item European Health Literacy Survey Questionnaire (HLS-EU Q16)*: 16-itm questionnaire to assess the health literacy pf the patient (54).

**References**

48. van der Molen T, Willemse BW, Schokker S, ten Hacken NH, Postma DS, Juniper EF. Development, validity and responsiveness of the Clinical COPD Questionnaire. Health Qual Life Outcomes. 2003;1:13.

49. Adams C, Walpola R, Schembri AM, Harrison R. The ultimate question? Evaluating the use of Net Promoter Score in healthcare: A systematic review. Health Expect. 2022;25(5):2328-39.

50. Hoogendoorn M, Jowett S, Dickens AP, Jordan R, Enocson A, Adab P, et al. Performance of the EQ-5D-5L Plus Respiratory Bolt-On in the Birmingham Chronic Obstructive Pulmonary Disease Cohort Study. Value Health. 2021;24(11):1667-75.

51. Hibbard JH, Stockard J, Mahoney ER, Tusler M. Development of the Patient Activation Measure (PAM): conceptualizing and measuring activation in patients and consumers. Health Serv Res. 2004;39(4 Pt 1):1005-26.

52. Lenferink A, Effing T, Harvey P, Battersby M, Frith P, van Beurden W, et al. Construct Validity of the Dutch Version of the 12-Item Partners in Health Scale: Measuring Patient Self-Management Behaviour and Knowledge in Patients with Chronic Obstructive Pulmonary Disease. PLoS One. 2016;11(8):e0161595.

53. Solem CT, Sun SX, Sudharshan L, Macahilig C, Katyal M, Gao X. Exacerbation-related impairment of quality of life and work productivity in severe and very severe chronic obstructive pulmonary disease. Int J Chron Obstruct Pulmon Dis. 2013;8:641-52.

54. Coenjaerds MMJ, Spruit MA, Cleutjens F, Ponds R, Franssen FME, Wouters EFM, et al. Health Literacy Among Patients With Chronic Lung Disease Entering Pulmonary Rehabilitation and Their Resident Loved Ones. J Cardiopulm Rehabil Prev. 2021;41(5):336-40.
